# Measurement of the sum of WW and WZ production with W+dijet events in pp collisions at $\sqrt{s} = 7\ \mbox{TeV}$

**DOI:** 10.1140/epjc/s10052-013-2283-3

**Published:** 2013-02-08

**Authors:** The CMS Collaboration, S. Chatrchyan, V. Khachatryan, A. M. Sirunyan, A. Tumasyan, W. Adam, E. Aguilo, T. Bergauer, M. Dragicevic, J. Erö, C. Fabjan, M. Friedl, R. Frühwirth, V. M. Ghete, J. Hammer, N. Hörmann, J. Hrubec, M. Jeitler, W. Kiesenhofer, V. Knünz, M. Krammer, I. Krätschmer, D. Liko, I. Mikulec, M. Pernicka, B. Rahbaran, C. Rohringer, H. Rohringer, R. Schöfbeck, J. Strauss, A. Taurok, W. Waltenberger, G. Walzel, C.-E. Wulz, V. Mossolov, N. Shumeiko, J. Suarez Gonzalez, M. Bansal, S. Bansal, T. Cornelis, E. A. De Wolf, X. Janssen, S. Luyckx, L. Mucibello, S. Ochesanu, B. Roland, R. Rougny, M. Selvaggi, H. Van Haevermaet, P. Van Mechelen, N. Van Remortel, A. Van Spilbeeck, F. Blekman, S. Blyweert, J. D’Hondt, R. Gonzalez Suarez, A. Kalogeropoulos, M. Maes, A. Olbrechts, W. Van Doninck, P. Van Mulders, G. P. Van Onsem, I. Villella, B. Clerbaux, G. De Lentdecker, V. Dero, A. P. R. Gay, T. Hreus, A. Léonard, P. E. Marage, A. Mohammadi, T. Reis, L. Thomas, C. Vander Velde, P. Vanlaer, J. Wang, V. Adler, K. Beernaert, A. Cimmino, S. Costantini, G. Garcia, M. Grunewald, B. Klein, J. Lellouch, A. Marinov, J. Mccartin, A. A. Ocampo Rios, D. Ryckbosch, N. Strobbe, F. Thyssen, M. Tytgat, S. Walsh, E. Yazgan, N. Zaganidis, S. Basegmez, G. Bruno, R. Castello, L. Ceard, C. Delaere, T. du Pree, D. Favart, L. Forthomme, A. Giammanco, J. Hollar, V. Lemaitre, J. Liao, O. Militaru, C. Nuttens, D. Pagano, A. Pin, K. Piotrzkowski, N. Schul, J. M. Vizan Garcia, N. Beliy, T. Caebergs, E. Daubie, G. H. Hammad, G. A. Alves, M. Correa Martins Junior, T. Martins, M. E. Pol, M. H. G. Souza, W. L. Aldá Júnior, W. Carvalho, A. Custódio, E. M. Da Costa, D. De Jesus Damiao, C. De Oliveira Martins, S. Fonseca De Souza, H. Malbouisson, M. Malek, D. Matos Figueiredo, L. Mundim, H. Nogima, W. L. Prado Da Silva, A. Santoro, L. Soares Jorge, A. Sznajder, A. Vilela Pereira, T. S. Anjos, C. A. Bernardes, F. A. Dias, T. R. Fernandez Perez Tomei, E. M. Gregores, C. Lagana, F. Marinho, P. G. Mercadante, S. F. Novaes, Sandra S. Padula, V. Genchev, P. Iaydjiev, S. Piperov, M. Rodozov, S. Stoykova, G. Sultanov, V. Tcholakov, R. Trayanov, M. Vutova, A. Dimitrov, R. Hadjiiska, V. Kozhuharov, L. Litov, B. Pavlov, P. Petkov, J. G. Bian, G. M. Chen, H. S. Chen, C. H. Jiang, D. Liang, S. Liang, X. Meng, J. Tao, J. Wang, X. Wang, Z. Wang, H. Xiao, M. Xu, J. Zang, Z. Zhang, C. Asawatangtrakuldee, Y. Ban, Y. Guo, W. Li, S. Liu, Y. Mao, S. J. Qian, H. Teng, D. Wang, L. Zhang, W. Zou, C. Avila, J. P. Gomez, B. Gomez Moreno, A. F. Osorio Oliveros, J. C. Sanabria, N. Godinovic, D. Lelas, R. Plestina, D. Polic, I. Puljak, Z. Antunovic, M. Kovac, V. Brigljevic, S. Duric, K. Kadija, J. Luetic, D. Mekterovic, S. Morovic, A. Attikis, M. Galanti, G. Mavromanolakis, J. Mousa, C. Nicolaou, F. Ptochos, P. A. Razis, M. Finger, M. Finger, Y. Assran, S. Elgammal, A. Ellithi Kamel, M. A. Mahmoud, A. Radi, M. Kadastik, M. Müntel, M. Raidal, L. Rebane, A. Tiko, P. Eerola, G. Fedi, M. Voutilainen, J. Härkönen, A. Heikkinen, V. Karimäki, R. Kinnunen, M. J. Kortelainen, T. Lampén, K. Lassila-Perini, S. Lehti, T. Lindén, P. Luukka, T. Mäenpää, T. Peltola, E. Tuominen, J. Tuominiemi, E. Tuovinen, D. Ungaro, L. Wendland, K. Banzuzi, A. Karjalainen, A. Korpela, T. Tuuva, M. Besancon, S. Choudhury, M. Dejardin, D. Denegri, B. Fabbro, J. L. Faure, F. Ferri, S. Ganjour, A. Givernaud, P. Gras, G. Hamel de Monchenault, P. Jarry, E. Locci, J. Malcles, L. Millischer, A. Nayak, J. Rander, A. Rosowsky, I. Shreyber, M. Titov, S. Baffioni, F. Beaudette, L. Benhabib, L. Bianchini, M. Bluj, C. Broutin, P. Busson, C. Charlot, N. Daci, T. Dahms, M. Dalchenko, L. Dobrzynski, A. Florent, R. Granier de Cassagnac, M. Haguenauer, P. Miné, C. Mironov, I. N. Naranjo, M. Nguyen, C. Ochando, P. Paganini, D. Sabes, R. Salerno, Y. Sirois, C. Veelken, A. Zabi, J.-L. Agram, J. Andrea, D. Bloch, D. Bodin, J.-M. Brom, M. Cardaci, E. C. Chabert, C. Collard, E. Conte, F. Drouhin, J.-C. Fontaine, D. Gelé, U. Goerlach, P. Juillot, A.-C. Le Bihan, P. Van Hove, F. Fassi, D. Mercier, S. Beauceron, N. Beaupere, O. Bondu, G. Boudoul, J. Chasserat, R. Chierici, D. Contardo, P. Depasse, H. El Mamouni, J. Fay, S. Gascon, M. Gouzevitch, B. Ille, T. Kurca, M. Lethuillier, L. Mirabito, S. Perries, L. Sgandurra, V. Sordini, Y. Tschudi, P. Verdier, S. Viret, Z. Tsamalaidze, C. Autermann, S. Beranek, B. Calpas, M. Edelhoff, L. Feld, N. Heracleous, O. Hindrichs, R. Jussen, K. Klein, J. Merz, A. Ostapchuk, A. Perieanu, F. Raupach, J. Sammet, S. Schael, D. Sprenger, H. Weber, B. Wittmer, V. Zhukov, M. Ata, J. Caudron, E. Dietz-Laursonn, D. Duchardt, M. Erdmann, R. Fischer, A. Güth, T. Hebbeker, C. Heidemann, K. Hoepfner, D. Klingebiel, P. Kreuzer, M. Merschmeyer, A. Meyer, M. Olschewski, P. Papacz, H. Pieta, H. Reithler, S. A. Schmitz, L. Sonnenschein, J. Steggemann, D. Teyssier, S. Thüer, M. Weber, M. Bontenackels, V. Cherepanov, Y. Erdogan, G. Flügge, H. Geenen, M. Geisler, W. Haj Ahmad, F. Hoehle, B. Kargoll, T. Kress, Y. Kuessel, J. Lingemann, A. Nowack, L. Perchalla, O. Pooth, P. Sauerland, A. Stahl, M. Aldaya Martin, J. Behr, W. Behrenhoff, U. Behrens, M. Bergholz, A. Bethani, K. Borras, A. Burgmeier, A. Cakir, L. Calligaris, A. Campbell, E. Castro, F. Costanza, D. Dammann, C. Diez Pardos, G. Eckerlin, D. Eckstein, G. Flucke, A. Geiser, I. Glushkov, P. Gunnellini, S. Habib, J. Hauk, G. Hellwig, H. Jung, M. Kasemann, P. Katsas, C. Kleinwort, H. Kluge, A. Knutsson, M. Krämer, D. Krücker, E. Kuznetsova, W. Lange, W. Lohmann, B. Lutz, R. Mankel, I. Marfin, M. Marienfeld, I.-A. Melzer-Pellmann, A. B. Meyer, J. Mnich, A. Mussgiller, S. Naumann-Emme, O. Novgorodova, J. Olzem, H. Perrey, A. Petrukhin, D. Pitzl, A. Raspereza, P. M. Ribeiro Cipriano, C. Riedl, E. Ron, M. Rosin, J. Salfeld-Nebgen, R. Schmidt, T. Schoerner-Sadenius, N. Sen, A. Spiridonov, M. Stein, R. Walsh, C. Wissing, V. Blobel, J. Draeger, H. Enderle, J. Erfle, U. Gebbert, M. Görner, T. Hermanns, R. S. Höing, K. Kaschube, G. Kaussen, H. Kirschenmann, R. Klanner, J. Lange, B. Mura, F. Nowak, T. Peiffer, N. Pietsch, D. Rathjens, C. Sander, H. Schettler, P. Schleper, E. Schlieckau, A. Schmidt, M. Schröder, T. Schum, M. Seidel, J. Sibille, V. Sola, H. Stadie, G. Steinbrück, J. Thomsen, L. Vanelderen, C. Barth, J. Berger, C. Böser, T. Chwalek, W. De Boer, A. Descroix, A. Dierlamm, M. Feindt, M. Guthoff, C. Hackstein, F. Hartmann, T. Hauth, M. Heinrich, H. Held, K. H. Hoffmann, U. Husemann, I. Katkov, J. R. Komaragiri, P. Lobelle Pardo, D. Martschei, S. Mueller, Th. Müller, M. Niegel, A. Nürnberg, O. Oberst, A. Oehler, J. Ott, G. Quast, K. Rabbertz, F. Ratnikov, N. Ratnikova, S. Röcker, F.-P. Schilling, G. Schott, H. J. Simonis, F. M. Stober, D. Troendle, R. Ulrich, J. Wagner-Kuhr, S. Wayand, T. Weiler, M. Zeise, G. Anagnostou, G. Daskalakis, T. Geralis, S. Kesisoglou, A. Kyriakis, D. Loukas, I. Manolakos, A. Markou, C. Markou, C. Mavrommatis, E. Ntomari, L. Gouskos, T. J. Mertzimekis, A. Panagiotou, N. Saoulidou, I. Evangelou, C. Foudas, P. Kokkas, N. Manthos, I. Papadopoulos, V. Patras, G. Bencze, C. Hajdu, P. Hidas, D. Horvath, F. Sikler, V. Veszpremi, G. Vesztergombi, N. Beni, S. Czellar, J. Molnar, J. Palinkas, Z. Szillasi, J. Karancsi, P. Raics, Z. L. Trocsanyi, B. Ujvari, S. B. Beri, V. Bhatnagar, N. Dhingra, R. Gupta, M. Kaur, M. Z. Mehta, N. Nishu, L. K. Saini, A. Sharma, J. B. Singh, A. Kumar, A. Kumar, S. Ahuja, A. Bhardwaj, B. C. Choudhary, S. Malhotra, M. Naimuddin, K. Ranjan, V. Sharma, R. K. Shivpuri, S. Banerjee, S. Bhattacharya, S. Dutta, B. Gomber, Sa. Jain, Sh. Jain, R. Khurana, S. Sarkar, M. Sharan, A. Abdulsalam, D. Dutta, S. Kailas, V. Kumar, A. K. Mohanty, L. M. Pant, P. Shukla, T. Aziz, S. Ganguly, M. Guchait, M. Maity, G. Majumder, K. Mazumdar, G. B. Mohanty, B. Parida, K. Sudhakar, N. Wickramage, S. Banerjee, S. Dugad, H. Arfaei, H. Bakhshiansohi, S. M. Etesami, A. Fahim, M. Hashemi, H. Hesari, A. Jafari, M. Khakzad, M. Mohammadi Najafabadi, S. Paktinat Mehdiabadi, B. Safarzadeh, M. Zeinali, M. Abbrescia, L. Barbone, C. Calabria, S. S. Chhibra, A. Colaleo, D. Creanza, N. De Filippis, M. De Palma, L. Fiore, G. Iaselli, G. Maggi, M. Maggi, B. Marangelli, S. My, S. Nuzzo, N. Pacifico, A. Pompili, G. Pugliese, G. Selvaggi, L. Silvestris, G. Singh, R. Venditti, P. Verwilligen, G. Zito, G. Abbiendi, A. C. Benvenuti, D. Bonacorsi, S. Braibant-Giacomelli, L. Brigliadori, P. Capiluppi, A. Castro, F. R. Cavallo, M. Cuffiani, G. M. Dallavalle, F. Fabbri, A. Fanfani, D. Fasanella, P. Giacomelli, C. Grandi, L. Guiducci, S. Marcellini, G. Masetti, M. Meneghelli, A. Montanari, F. L. Navarria, F. Odorici, A. Perrotta, F. Primavera, A. M. Rossi, T. Rovelli, G. P. Siroli, N. Tosi, R. Travaglini, S. Albergo, G. Cappello, M. Chiorboli, S. Costa, R. Potenza, A. Tricomi, C. Tuve, G. Barbagli, V. Ciulli, C. Civinini, R. D’Alessandro, E. Focardi, S. Frosali, E. Gallo, S. Gonzi, M. Meschini, S. Paoletti, G. Sguazzoni, A. Tropiano, L. Benussi, S. Bianco, S. Colafranceschi, F. Fabbri, D. Piccolo, P. Fabbricatore, R. Musenich, S. Tosi, A. Benaglia, F. De Guio, L. Di Matteo, S. Fiorendi, S. Gennai, A. Ghezzi, S. Malvezzi, R. A. Manzoni, A. Martelli, A. Massironi, D. Menasce, L. Moroni, M. Paganoni, D. Pedrini, S. Ragazzi, N. Redaelli, S. Sala, T. Tabarelli de Fatis, S. Buontempo, C. A. Carrillo Montoya, N. Cavallo, A. De Cosa, O. Dogangun, F. Fabozzi, A. O. M. Iorio, L. Lista, S. Meola, M. Merola, P. Paolucci, P. Azzi, N. Bacchetta, D. Bisello, A. Branca, R. Carlin, P. Checchia, T. Dorigo, F. Gasparini, U. Gasparini, A. Gozzelino, K. Kanishchev, S. Lacaprara, I. Lazzizzera, M. Margoni, A. T. Meneguzzo, J. Pazzini, N. Pozzobon, P. Ronchese, F. Simonetto, E. Torassa, M. Tosi, S. Vanini, S. Ventura, P. Zotto, G. Zumerle, M. Gabusi, S. P. Ratti, C. Riccardi, P. Torre, P. Vitulo, M. Biasini, G. M. Bilei, L. Fanò, P. Lariccia, G. Mantovani, M. Menichelli, A. Nappi, F. Romeo, A. Saha, A. Santocchia, A. Spiezia, S. Taroni, P. Azzurri, G. Bagliesi, J. Bernardini, T. Boccali, G. Broccolo, R. Castaldi, R. T. D’Agnolo, R. Dell’Orso, F. Fiori, L. Foà, A. Giassi, A. Kraan, F. Ligabue, T. Lomtadze, L. Martini, A. Messineo, F. Palla, A. Rizzi, A. T. Serban, P. Spagnolo, P. Squillacioti, R. Tenchini, G. Tonelli, A. Venturi, P. G. Verdini, L. Barone, F. Cavallari, D. Del Re, M. Diemoz, C. Fanelli, M. Grassi, E. Longo, P. Meridiani, F. Micheli, S. Nourbakhsh, G. Organtini, R. Paramatti, S. Rahatlou, M. Sigamani, L. Soffi, N. Amapane, R. Arcidiacono, S. Argiro, M. Arneodo, C. Biino, N. Cartiglia, S. Casasso, M. Costa, N. Demaria, C. Mariotti, S. Maselli, E. Migliore, V. Monaco, M. Musich, M. M. Obertino, N. Pastrone, M. Pelliccioni, A. Potenza, A. Romero, M. Ruspa, R. Sacchi, A. Solano, A. Staiano, S. Belforte, V. Candelise, M. Casarsa, F. Cossutti, G. Della Ricca, B. Gobbo, M. Marone, D. Montanino, A. Penzo, A. Schizzi, T. Y. Kim, S. K. Nam, S. Chang, D. H. Kim, G. N. Kim, D. J. Kong, H. Park, S. R. Ro, D. C. Son, T. Son, J. Y. Kim, Zero J. Kim, S. Song, S. Choi, D. Gyun, B. Hong, M. Jo, H. Kim, T. J. Kim, K. S. Lee, D. H. Moon, S. K. Park, M. Choi, J. H. Kim, C. Park, I. C. Park, S. Park, G. Ryu, Y. Choi, Y. K. Choi, J. Goh, M. S. Kim, E. Kwon, B. Lee, J. Lee, S. Lee, H. Seo, I. Yu, M. J. Bilinskas, I. Grigelionis, M. Janulis, A. Juodagalvis, H. Castilla-Valdez, E. De La Cruz-Burelo, I. Heredia-de La Cruz, R. Lopez-Fernandez, R. Magaña Villalba, J. Martínez-Ortega, A. Sánchez-Hernández, L. M. Villasenor-Cendejas, S. Carrillo Moreno, F. Vazquez Valencia, H. A. Salazar Ibarguen, E. Casimiro Linares, A. Morelos Pineda, M. A. Reyes-Santos, D. Krofcheck, A. J. Bell, P. H. Butler, R. Doesburg, S. Reucroft, H. Silverwood, M. Ahmad, M. I. Asghar, J. Butt, H. R. Hoorani, S. Khalid, W. A. Khan, T. Khurshid, S. Qazi, M. A. Shah, M. Shoaib, H. Bialkowska, B. Boimska, T. Frueboes, R. Gokieli, M. Górski, M. Kazana, K. Nawrocki, K. Romanowska-Rybinska, M. Szleper, G. Wrochna, P. Zalewski, G. Brona, K. Bunkowski, M. Cwiok, W. Dominik, K. Doroba, A. Kalinowski, M. Konecki, J. Krolikowski, N. Almeida, P. Bargassa, A. David, P. Faccioli, P. G. Ferreira Parracho, M. Gallinaro, J. Seixas, J. Varela, P. Vischia, P. Bunin, M. Gavrilenko, I. Golutvin, I. Gorbunov, V. Karjavin, V. Konoplyanikov, G. Kozlov, A. Lanev, A. Malakhov, P. Moisenz, V. Palichik, V. Perelygin, S. Shmatov, S. Shulha, V. Smirnov, A. Volodko, A. Zarubin, S. Evstyukhin, V. Golovtsov, Y. Ivanov, V. Kim, P. Levchenko, V. Murzin, V. Oreshkin, I. Smirnov, V. Sulimov, L. Uvarov, S. Vavilov, A. Vorobyev, An. Vorobyev, Yu. Andreev, A. Dermenev, S. Gninenko, N. Golubev, M. Kirsanov, N. Krasnikov, V. Matveev, A. Pashenkov, D. Tlisov, A. Toropin, V. Epshteyn, M. Erofeeva, V. Gavrilov, M. Kossov, N. Lychkovskaya, V. Popov, G. Safronov, S. Semenov, V. Stolin, E. Vlasov, A. Zhokin, A. Belyaev, E. Boos, M. Dubinin, L. Dudko, A. Ershov, A. Gribushin, V. Klyukhin, O. Kodolova, I. Lokhtin, A. Markina, S. Obraztsov, M. Perfilov, S. Petrushanko, A. Popov, L. Sarycheva, V. Savrin, A. Snigirev, V. Andreev, M. Azarkin, I. Dremin, M. Kirakosyan, A. Leonidov, G. Mesyats, S. V. Rusakov, A. Vinogradov, I. Azhgirey, I. Bayshev, S. Bitioukov, V. Grishin, V. Kachanov, D. Konstantinov, V. Krychkine, V. Petrov, R. Ryutin, A. Sobol, L. Tourtchanovitch, S. Troshin, N. Tyurin, A. Uzunian, A. Volkov, P. Adzic, M. Djordjevic, M. Ekmedzic, D. Krpic, J. Milosevic, M. Aguilar-Benitez, J. Alcaraz Maestre, P. Arce, C. Battilana, E. Calvo, M. Cerrada, M. Chamizo Llatas, N. Colino, B. De La Cruz, A. Delgado Peris, D. Domínguez Vázquez, C. Fernandez Bedoya, J. P. Fernández Ramos, A. Ferrando, J. Flix, M. C. Fouz, P. Garcia-Abia, O. Gonzalez Lopez, S. Goy Lopez, J. M. Hernandez, M. I. Josa, G. Merino, J. Puerta Pelayo, A. Quintario Olmeda, I. Redondo, L. Romero, J. Santaolalla, M. S. Soares, C. Willmott, C. Albajar, G. Codispoti, J. F. de Trocóniz, H. Brun, J. Cuevas, J. Fernandez Menendez, S. Folgueras, I. Gonzalez Caballero, L. Lloret Iglesias, J. Piedra Gomez, J. A. Brochero Cifuentes, I. J. Cabrillo, A. Calderon, S. H. Chuang, J. Duarte Campderros, M. Felcini, M. Fernandez, G. Gomez, J. Gonzalez Sanchez, A. Graziano, C. Jorda, A. Lopez Virto, J. Marco, R. Marco, C. Martinez Rivero, F. Matorras, F. J. Munoz Sanchez, T. Rodrigo, A. Y. Rodríguez-Marrero, A. Ruiz-Jimeno, L. Scodellaro, I. Vila, R. Vilar Cortabitarte, D. Abbaneo, E. Auffray, G. Auzinger, M. Bachtis, P. Baillon, A. H. Ball, D. Barney, J. F. Benitez, C. Bernet, G. Bianchi, P. Bloch, A. Bocci, A. Bonato, C. Botta, H. Breuker, T. Camporesi, G. Cerminara, T. Christiansen, J. A. Coarasa Perez, D. D’Enterria, A. Dabrowski, A. De Roeck, S. Di Guida, M. Dobson, N. Dupont-Sagorin, A. Elliott-Peisert, B. Frisch, W. Funk, G. Georgiou, M. Giffels, D. Gigi, K. Gill, D. Giordano, M. Girone, M. Giunta, F. Glege, R. Gomez-Reino Garrido, P. Govoni, S. Gowdy, R. Guida, M. Hansen, P. Harris, C. Hartl, J. Harvey, B. Hegner, A. Hinzmann, V. Innocente, P. Janot, K. Kaadze, E. Karavakis, K. Kousouris, P. Lecoq, Y.-J. Lee, P. Lenzi, C. Lourenço, N. Magini, T. Mäki, M. Malberti, L. Malgeri, M. Mannelli, L. Masetti, F. Meijers, S. Mersi, E. Meschi, R. Moser, M. U. Mozer, M. Mulders, P. Musella, E. Nesvold, T. Orimoto, L. Orsini, E. Palencia Cortezon, E. Perez, L. Perrozzi, A. Petrilli, A. Pfeiffer, M. Pierini, M. Pimiä, D. Piparo, G. Polese, L. Quertenmont, A. Racz, W. Reece, J. Rodrigues Antunes, G. Rolandi, C. Rovelli, M. Rovere, H. Sakulin, F. Santanastasio, C. Schäfer, C. Schwick, I. Segoni, S. Sekmen, A. Sharma, P. Siegrist, P. Silva, M. Simon, P. Sphicas, D. Spiga, A. Tsirou, G. I. Veres, J. R. Vlimant, H. K. Wöhri, S. D. Worm, W. D. Zeuner, W. Bertl, K. Deiters, W. Erdmann, K. Gabathuler, R. Horisberger, Q. Ingram, H. C. Kaestli, S. König, D. Kotlinski, U. Langenegger, F. Meier, D. Renker, T. Rohe, L. Bäni, P. Bortignon, M. A. Buchmann, B. Casal, N. Chanon, A. Deisher, G. Dissertori, M. Dittmar, M. Donegà, M. Dünser, J. Eugster, K. Freudenreich, C. Grab, D. Hits, P. Lecomte, W. Lustermann, A. C. Marini, P. Martinez Ruiz del Arbol, N. Mohr, F. Moortgat, C. Nägeli, P. Nef, F. Nessi-Tedaldi, F. Pandolfi, L. Pape, F. Pauss, M. Peruzzi, F. J. Ronga, M. Rossini, L. Sala, A. K. Sanchez, A. Starodumov, B. Stieger, M. Takahashi, L. Tauscher, A. Thea, K. Theofilatos, D. Treille, C. Urscheler, R. Wallny, H. A. Weber, L. Wehrli, C. Amsler, V. Chiochia, S. De Visscher, C. Favaro, M. Ivova Rikova, B. Kilminster, B. Millan Mejias, P. Otiougova, P. Robmann, H. Snoek, S. Tupputi, M. Verzetti, Y. H. Chang, K. H. Chen, C. Ferro, C. M. Kuo, S. W. Li, W. Lin, Y. J. Lu, A. P. Singh, R. Volpe, S. S. Yu, P. Bartalini, P. Chang, Y. H. Chang, Y. W. Chang, Y. Chao, K. F. Chen, C. Dietz, U. Grundler, W.-S. Hou, Y. Hsiung, K. Y. Kao, Y. J. Lei, R.-S. Lu, D. Majumder, E. Petrakou, X. Shi, J. G. Shiu, Y. M. Tzeng, X. Wan, M. Wang, B. Asavapibhop, N. Srimanobhas, A. Adiguzel, M. N. Bakirci, S. Cerci, C. Dozen, I. Dumanoglu, E. Eskut, S. Girgis, G. Gokbulut, E. Gurpinar, I. Hos, E. E. Kangal, T. Karaman, G. Karapinar, A. Kayis Topaksu, G. Onengut, K. Ozdemir, S. Ozturk, A. Polatoz, K. Sogut, D. Sunar Cerci, B. Tali, H. Topakli, L. N. Vergili, M. Vergili, I. V. Akin, T. Aliev, B. Bilin, S. Bilmis, M. Deniz, H. Gamsizkan, A. M. Guler, K. Ocalan, A. Ozpineci, M. Serin, R. Sever, U. E. Surat, M. Yalvac, E. Yildirim, M. Zeyrek, E. Gülmez, B. Isildak, M. Kaya, O. Kaya, S. Ozkorucuklu, N. Sonmez, K. Cankocak, L. Levchuk, J. J. Brooke, E. Clement, D. Cussans, H. Flacher, R. Frazier, J. Goldstein, M. Grimes, G. P. Heath, H. F. Heath, L. Kreczko, S. Metson, D. M. Newbold, K. Nirunpong, A. Poll, S. Senkin, V. J. Smith, T. Williams, L. Basso, K. W. Bell, A. Belyaev, C. Brew, R. M. Brown, D. J. A. Cockerill, J. A. Coughlan, K. Harder, S. Harper, J. Jackson, B. W. Kennedy, E. Olaiya, D. Petyt, B. C. Radburn-Smith, C. H. Shepherd-Themistocleous, I. R. Tomalin, W. J. Womersley, R. Bainbridge, G. Ball, R. Beuselinck, O. Buchmuller, D. Colling, N. Cripps, M. Cutajar, P. Dauncey, G. Davies, M. Della Negra, W. Ferguson, J. Fulcher, D. Futyan, A. Gilbert, A. Guneratne Bryer, G. Hall, Z. Hatherell, J. Hays, G. Iles, M. Jarvis, G. Karapostoli, L. Lyons, A.-M. Magnan, J. Marrouche, B. Mathias, R. Nandi, J. Nash, A. Nikitenko, A. Papageorgiou, J. Pela, M. Pesaresi, K. Petridis, M. Pioppi, D. M. Raymond, S. Rogerson, A. Rose, M. J. Ryan, C. Seez, P. Sharp, A. Sparrow, M. Stoye, A. Tapper, M. Vazquez Acosta, T. Virdee, S. Wakefield, N. Wardle, T. Whyntie, M. Chadwick, J. E. Cole, P. R. Hobson, A. Khan, P. Kyberd, D. Leggat, D. Leslie, W. Martin, I. D. Reid, P. Symonds, L. Teodorescu, M. Turner, K. Hatakeyama, H. Liu, T. Scarborough, O. Charaf, C. Henderson, P. Rumerio, A. Avetisyan, T. Bose, C. Fantasia, A. Heister, J. St. John, P. Lawson, D. Lazic, J. Rohlf, D. Sperka, L. Sulak, J. Alimena, S. Bhattacharya, G. Christopher, D. Cutts, Z. Demiragli, A. Ferapontov, A. Garabedian, U. Heintz, S. Jabeen, G. Kukartsev, E. Laird, G. Landsberg, M. Luk, M. Narain, D. Nguyen, M. Segala, T. Sinthuprasith, T. Speer, R. Breedon, G. Breto, M. Calderon De La Barca Sanchez, S. Chauhan, M. Chertok, J. Conway, R. Conway, P. T. Cox, J. Dolen, R. Erbacher, M. Gardner, R. Houtz, W. Ko, A. Kopecky, R. Lander, O. Mall, T. Miceli, D. Pellett, F. Ricci-Tam, B. Rutherford, M. Searle, J. Smith, M. Squires, M. Tripathi, R. Vasquez Sierra, R. Yohay, V. Andreev, D. Cline, R. Cousins, J. Duris, S. Erhan, P. Everaerts, C. Farrell, J. Hauser, M. Ignatenko, C. Jarvis, G. Rakness, P. Schlein, P. Traczyk, V. Valuev, M. Weber, J. Babb, R. Clare, M. E. Dinardo, J. Ellison, J. W. Gary, F. Giordano, G. Hanson, G. Y. Jeng, H. Liu, O. R. Long, A. Luthra, H. Nguyen, S. Paramesvaran, J. Sturdy, S. Sumowidagdo, R. Wilken, S. Wimpenny, W. Andrews, J. G. Branson, G. B. Cerati, S. Cittolin, D. Evans, A. Holzner, R. Kelley, M. Lebourgeois, J. Letts, I. Macneill, B. Mangano, S. Padhi, C. Palmer, G. Petrucciani, M. Pieri, M. Sani, V. Sharma, S. Simon, E. Sudano, M. Tadel, Y. Tu, A. Vartak, S. Wasserbaech, F. Würthwein, A. Yagil, J. Yoo, D. Barge, R. Bellan, C. Campagnari, M. D’Alfonso, T. Danielson, K. Flowers, P. Geffert, F. Golf, J. Incandela, C. Justus, P. Kalavase, D. Kovalskyi, V. Krutelyov, S. Lowette, N. Mccoll, V. Pavlunin, J. Ribnik, J. Richman, R. Rossin, D. Stuart, W. To, C. West, A. Apresyan, A. Bornheim, Y. Chen, E. Di Marco, J. Duarte, M. Gataullin, Y. Ma, A. Mott, H. B. Newman, C. Rogan, M. Spiropulu, V. Timciuc, J. Veverka, R. Wilkinson, S. Xie, Y. Yang, R. Y. Zhu, V. Azzolini, A. Calamba, R. Carroll, T. Ferguson, Y. Iiyama, D. W. Jang, Y. F. Liu, M. Paulini, H. Vogel, I. Vorobiev, J. P. Cumalat, B. R. Drell, W. T. Ford, A. Gaz, E. Luiggi Lopez, J. G. Smith, K. Stenson, K. A. Ulmer, S. R. Wagner, J. Alexander, A. Chatterjee, N. Eggert, L. K. Gibbons, B. Heltsley, A. Khukhunaishvili, B. Kreis, N. Mirman, G. Nicolas Kaufman, J. R. Patterson, A. Ryd, E. Salvati, W. Sun, W. D. Teo, J. Thom, J. Thompson, J. Tucker, J. Vaughan, Y. Weng, L. Winstrom, P. Wittich, D. Winn, S. Abdullin, M. Albrow, J. Anderson, G. Apollinari, L. A. T. Bauerdick, A. Beretvas, J. Berryhill, P. C. Bhat, K. Burkett, J. N. Butler, V. Chetluru, H. W. K. Cheung, F. Chlebana, V. D. Elvira, I. Fisk, J. Freeman, Y. Gao, D. Green, O. Gutsche, J. Hanlon, R. M. Harris, J. Hirschauer, B. Hooberman, S. Jindariani, M. Johnson, U. Joshi, B. Klima, S. Kunori, S. Kwan, C. Leonidopoulos, J. Linacre, D. Lincoln, R. Lipton, J. Lykken, K. Maeshima, J. M. Marraffino, S. Maruyama, D. Mason, P. McBride, K. Mishra, S. Mrenna, Y. Musienko, C. Newman-Holmes, V. O’Dell, E. Sexton-Kennedy, S. Sharma, W. J. Spalding, L. Spiegel, L. Taylor, S. Tkaczyk, N. V. Tran, L. Uplegger, E. W. Vaandering, R. Vidal, J. Whitmore, W. Wu, F. Yang, J. C. Yun, D. Acosta, P. Avery, D. Bourilkov, M. Chen, T. Cheng, S. Das, M. De Gruttola, G. P. Di Giovanni, D. Dobur, A. Drozdetskiy, R. D. Field, M. Fisher, Y. Fu, I. K. Furic, J. Gartner, J. Hugon, B. Kim, J. Konigsberg, A. Korytov, A. Kropivnitskaya, T. Kypreos, J. F. Low, K. Matchev, P. Milenovic, G. Mitselmakher, L. Muniz, M. Park, R. Remington, A. Rinkevicius, P. Sellers, N. Skhirtladze, M. Snowball, J. Yelton, M. Zakaria, V. Gaultney, S. Hewamanage, L. M. Lebolo, S. Linn, P. Markowitz, G. Martinez, J. L. Rodriguez, T. Adams, A. Askew, J. Bochenek, J. Chen, B. Diamond, S. V. Gleyzer, J. Haas, S. Hagopian, V. Hagopian, M. Jenkins, K. F. Johnson, H. Prosper, V. Veeraraghavan, M. Weinberg, M. M. Baarmand, B. Dorney, M. Hohlmann, H. Kalakhety, I. Vodopiyanov, F. Yumiceva, M. R. Adams, I. M. Anghel, L. Apanasevich, Y. Bai, V. E. Bazterra, R. R. Betts, I. Bucinskaite, J. Callner, R. Cavanaugh, O. Evdokimov, L. Gauthier, C. E. Gerber, D. J. Hofman, S. Khalatyan, F. Lacroix, C. O’Brien, C. Silkworth, D. Strom, P. Turner, N. Varelas, U. Akgun, E. A. Albayrak, B. Bilki, W. Clarida, F. Duru, J.-P. Merlo, H. Mermerkaya, A. Mestvirishvili, A. Moeller, J. Nachtman, C. R. Newsom, E. Norbeck, Y. Onel, F. Ozok, S. Sen, P. Tan, E. Tiras, J. Wetzel, T. Yetkin, K. Yi, B. A. Barnett, B. Blumenfeld, S. Bolognesi, D. Fehling, G. Giurgiu, A. V. Gritsan, Z. J. Guo, G. Hu, P. Maksimovic, M. Swartz, A. Whitbeck, P. Baringer, A. Bean, G. Benelli, R. P. Kenny Iii, M. Murray, D. Noonan, S. Sanders, R. Stringer, G. Tinti, J. S. Wood, A. F. Barfuss, T. Bolton, I. Chakaberia, A. Ivanov, S. Khalil, M. Makouski, Y. Maravin, S. Shrestha, I. Svintradze, J. Gronberg, D. Lange, F. Rebassoo, D. Wright, A. Baden, B. Calvert, S. C. Eno, J. A. Gomez, N. J. Hadley, R. G. Kellogg, M. Kirn, T. Kolberg, Y. Lu, M. Marionneau, A. C. Mignerey, K. Pedro, A. Skuja, J. Temple, M. B. Tonjes, S. C. Tonwar, E. Twedt, A. Apyan, G. Bauer, J. Bendavid, W. Busza, E. Butz, I. A. Cali, M. Chan, V. Dutta, G. Gomez Ceballos, M. Goncharov, K. A. Hahn, Y. Kim, M. Klute, K. Krajczar, P. D. Luckey, T. Ma, S. Nahn, C. Paus, D. Ralph, C. Roland, G. Roland, M. Rudolph, G. S. F. Stephans, F. Stöckli, K. Sumorok, K. Sung, D. Velicanu, E. A. Wenger, R. Wolf, B. Wyslouch, M. Yang, Y. Yilmaz, A. S. Yoon, M. Zanetti, V. Zhukova, S. I. Cooper, B. Dahmes, A. De Benedetti, G. Franzoni, A. Gude, S. C. Kao, K. Klapoetke, Y. Kubota, J. Mans, N. Pastika, R. Rusack, M. Sasseville, A. Singovsky, N. Tambe, J. Turkewitz, L. M. Cremaldi, R. Kroeger, L. Perera, R. Rahmat, D. A. Sanders, E. Avdeeva, K. Bloom, S. Bose, D. R. Claes, A. Dominguez, M. Eads, J. Keller, I. Kravchenko, J. Lazo-Flores, S. Malik, G. R. Snow, A. Godshalk, I. Iashvili, S. Jain, A. Kharchilava, K. Krylova, A. Kumar, S. Rappoccio, G. Alverson, E. Barberis, D. Baumgartel, M. Chasco, J. Haley, D. Nash, D. Trocino, D. Wood, J. Zhang, A. Anastassov, A. Kubik, L. Lusito, N. Mucia, N. Odell, R. A. Ofierzynski, B. Pollack, A. Pozdnyakov, R. Sarkar, M. Schmitt, S. Stoynev, M. Velasco, S. Won, L. Antonelli, D. Berry, A. Brinkerhoff, K. M. Chan, M. Hildreth, C. Jessop, D. J. Karmgard, J. Kolb, K. Lannon, W. Luo, S. Lynch, N. Marinelli, D. M. Morse, T. Pearson, M. Planer, R. Ruchti, J. Slaunwhite, N. Valls, M. Wayne, M. Wolf, B. Bylsma, L. S. Durkin, C. Hill, R. Hughes, K. Kotov, T. Y. Ling, D. Puigh, M. Rodenburg, C. Vuosalo, G. Williams, B. L. Winer, E. Berry, P. Elmer, V. Halyo, P. Hebda, J. Hegeman, A. Hunt, P. Jindal, S. A. Koay, D. Lopes Pegna, P. Lujan, D. Marlow, T. Medvedeva, M. Mooney, J. Olsen, P. Piroué, X. Quan, A. Raval, H. Saka, D. Stickland, C. Tully, J. S. Werner, A. Zuranski, E. Brownson, A. Lopez, H. Mendez, J. E. Ramirez Vargas, E. Alagoz, V. E. Barnes, D. Benedetti, G. Bolla, D. Bortoletto, M. De Mattia, A. Everett, Z. Hu, M. Jones, O. Koybasi, M. Kress, A. T. Laasanen, N. Leonardo, V. Maroussov, P. Merkel, D. H. Miller, N. Neumeister, I. Shipsey, D. Silvers, A. Svyatkovskiy, M. Vidal Marono, H. D. Yoo, J. Zablocki, Y. Zheng, S. Guragain, N. Parashar, A. Adair, B. Akgun, C. Boulahouache, K. M. Ecklund, F. J. M. Geurts, W. Li, B. P. Padley, R. Redjimi, J. Roberts, J. Zabel, B. Betchart, A. Bodek, Y. S. Chung, R. Covarelli, P. de Barbaro, R. Demina, Y. Eshaq, T. Ferbel, A. Garcia-Bellido, P. Goldenzweig, J. Han, A. Harel, D. C. Miner, D. Vishnevskiy, M. Zielinski, A. Bhatti, R. Ciesielski, L. Demortier, K. Goulianos, G. Lungu, S. Malik, C. Mesropian, S. Arora, A. Barker, J. P. Chou, C. Contreras-Campana, E. Contreras-Campana, D. Duggan, D. Ferencek, Y. Gershtein, R. Gray, E. Halkiadakis, D. Hidas, A. Lath, S. Panwalkar, M. Park, R. Patel, V. Rekovic, J. Robles, K. Rose, S. Salur, S. Schnetzer, C. Seitz, S. Somalwar, R. Stone, S. Thomas, M. Walker, G. Cerizza, M. Hollingsworth, S. Spanier, Z. C. Yang, A. York, R. Eusebi, W. Flanagan, J. Gilmore, T. Kamon, V. Khotilovich, R. Montalvo, I. Osipenkov, Y. Pakhotin, A. Perloff, J. Roe, A. Safonov, T. Sakuma, S. Sengupta, I. Suarez, A. Tatarinov, D. Toback, N. Akchurin, J. Damgov, C. Dragoiu, P. R. Dudero, C. Jeong, K. Kovitanggoon, S. W. Lee, T. Libeiro, Y. Roh, I. Volobouev, E. Appelt, A. G. Delannoy, C. Florez, S. Greene, A. Gurrola, W. Johns, P. Kurt, C. Maguire, A. Melo, M. Sharma, P. Sheldon, B. Snook, S. Tuo, J. Velkovska, M. W. Arenton, M. Balazs, S. Boutle, B. Cox, B. Francis, J. Goodell, R. Hirosky, A. Ledovskoy, C. Lin, C. Neu, J. Wood, S. Gollapinni, R. Harr, P. E. Karchin, C. Kottachchi Kankanamge Don, P. Lamichhane, K. Mcgee, A. Sakharov, K. Siehl, M. Anderson, D. Belknap, L. Borrello, D. Carlsmith, M. Cepeda, S. Dasu, E. Friis, L. Gray, K. S. Grogg, M. Grothe, R. Hall-Wilton, M. Herndon, A. Hervé, P. Klabbers, J. Klukas, A. Lanaro, C. Lazaridis, J. Leonard, R. Loveless, A. Mohapatra, I. Ojalvo, F. Palmonari, G. A. Pierro, I. Ross, A. Savin, W. H. Smith, J. Swanson

**Affiliations:** 1 CERN, Geneva, Switzerland; 2 Yerevan Physics Institute, Yerevan, Armenia; 3 Institut für Hochenergiephysik der OeAW, Wien, Austria; 4 National Centre for Particle and High Energy Physics, Minsk, Belarus; 5 Universiteit Antwerpen, Antwerpen, Belgium; 6 Vrije Universiteit Brussel, Brussel, Belgium; 7 Université Libre de Bruxelles, Bruxelles, Belgium; 8 Ghent University, Ghent, Belgium; 9 Université Catholique de Louvain, Louvain-la-Neuve, Belgium; 10 Université de Mons, Mons, Belgium; 11 Centro Brasileiro de Pesquisas Fisicas, Rio de Janeiro, Brazil; 12 Universidade do Estado do Rio de Janeiro, Rio de Janeiro, Brazil; 13 Instituto de Fisica Teorica, Universidade Estadual Paulista, Sao Paulo, Brazil; 14 Institute for Nuclear Research and Nuclear Energy, Sofia, Bulgaria; 15 University of Sofia, Sofia, Bulgaria; 16 Institute of High Energy Physics, Beijing, China; 17 State Key Lab. of Nucl. Phys. and Tech., Peking University, Beijing, China; 18 Universidad de Los Andes, Bogota, Colombia; 19 Technical University of Split, Split, Croatia; 20 University of Split, Split, Croatia; 21 Institute Rudjer Boskovic, Zagreb, Croatia; 22 University of Cyprus, Nicosia, Cyprus; 23 Charles University, Prague, Czech Republic; 24 Egyptian Network of High Energy Physics, Academy of Scientific Research and Technology of the Arab Republic of Egypt, Cairo, Egypt; 25 National Institute of Chemical Physics and Biophysics, Tallinn, Estonia; 26 Department of Physics, University of Helsinki, Helsinki, Finland; 27 Helsinki Institute of Physics, Helsinki, Finland; 28 Lappeenranta University of Technology, Lappeenranta, Finland; 29 DSM/IRFU, CEA/Saclay, Gif-sur-Yvette, France; 30 Laboratoire Leprince-Ringuet, Ecole Polytechnique, IN2P3-CNRS, Palaiseau, France; 31 Institut Pluridisciplinaire Hubert Curien, Université de Strasbourg, Université de Haute Alsace Mulhouse, CNRS/IN2P3, Strasbourg, France; 32 Centre de Calcul de l’Institut National de Physique Nucleaire et de Physique des Particules, CNRS/IN2P3, Villeurbanne, France; 33 CNRS-IN2P3, Institut de Physique Nucléaire de Lyon, Université de Lyon, Université Claude Bernard Lyon 1, Villeurbanne, France; 34 Institute of High Energy Physics and Informatization, Tbilisi State University, Tbilisi, Georgia; 35 I. Physikalisches Institut, RWTH Aachen University, Aachen, Germany; 36 III. Physikalisches Institut A, RWTH Aachen University, Aachen, Germany; 37 III. Physikalisches Institut B, RWTH Aachen University, Aachen, Germany; 38 Deutsches Elektronen-Synchrotron, Hamburg, Germany; 39 University of Hamburg, Hamburg, Germany; 40 Institut für Experimentelle Kernphysik, Karlsruhe, Germany; 41 Institute of Nuclear Physics “Demokritos”, Aghia Paraskevi, Greece; 42 University of Athens, Athens, Greece; 43 University of Ioánnina, Ioánnina, Greece; 44 KFKI Research Institute for Particle and Nuclear Physics, Budapest, Hungary; 45 Institute of Nuclear Research ATOMKI, Debrecen, Hungary; 46 University of Debrecen, Debrecen, Hungary; 47 Panjab University, Chandigarh, India; 48 University of Delhi, Delhi, India; 49 Saha Institute of Nuclear Physics, Kolkata, India; 50 Bhabha Atomic Research Centre, Mumbai, India; 51 Tata Institute of Fundamental Research - EHEP, Mumbai, India; 52 Tata Institute of Fundamental Research - HECR, Mumbai, India; 53 Institute for Research in Fundamental Sciences (IPM), Tehran, Iran; 54 INFN Sezione di Bari, Bari, Italy; 55 Università di Bari, Bari, Italy; 56 Politecnico di Bari, Bari, Italy; 57 INFN Sezione di Bologna, Bologna, Italy; 58 Università di Bologna, Bologna, Italy; 59 INFN Sezione di Catania, Catania, Italy; 60 Università di Catania, Catania, Italy; 61 INFN Sezione di Firenze, Firenze, Italy; 62 Università di Firenze, Firenze, Italy; 63 INFN Laboratori Nazionali di Frascati, Frascati, Italy; 64 INFN Sezione di Genova, Genova, Italy; 65 Università di Genova, Genova, Italy; 66 INFN Sezione di Milano-Bicocca, Milano, Italy; 67 Università di Milano-Bicocca, Milano, Italy; 68 INFN Sezione di Napoli, Napoli, Italy; 69 Università di Napoli “Federico II”, Napoli, Italy; 70 INFN Sezione di Padova, Padova, Italy; 71 Università di Padova, Padova, Italy; 72 Università di Trento (Trento), Padova, Italy; 73 INFN Sezione di Pavia, Pavia, Italy; 74 Università di Pavia, Pavia, Italy; 75 INFN Sezione di Perugia, Perugia, Italy; 76 Università di Perugia, Perugia, Italy; 77 INFN Sezione di Pisa, Pisa, Italy; 78 Università di Pisa, Pisa, Italy; 79 Scuola Normale Superiore di Pisa, Pisa, Italy; 80 INFN Sezione di Roma, Roma, Italy; 81 Università di Roma, Roma, Italy; 82 INFN Sezione di Torino, Torino, Italy; 83 Università di Torino, Torino, Italy; 84 Università del Piemonte Orientale (Novara), Torino, Italy; 85 INFN Sezione di Trieste, Trieste, Italy; 86 Università di Trieste, Trieste, Italy; 87 Kangwon National University, Chunchon, Korea; 88 Kyungpook National University, Daegu, Korea; 89 Institute for Universe and Elementary Particles, Chonnam National University, Kwangju, Korea; 90 Korea University, Seoul, Korea; 91 University of Seoul, Seoul, Korea; 92 Sungkyunkwan University, Suwon, Korea; 93 Vilnius University, Vilnius, Lithuania; 94 Centro de Investigacion y de Estudios Avanzados del IPN, Mexico City, Mexico; 95 Universidad Iberoamericana, Mexico City, Mexico; 96 Benemerita Universidad Autonoma de Puebla, Puebla, Mexico; 97 Universidad Autónoma de San Luis Potosí, San Luis Potosí, Mexico; 98 University of Auckland, Auckland, New Zealand; 99 University of Canterbury, Christchurch, New Zealand; 100 National Centre for Physics, Quaid-I-Azam University, Islamabad, Pakistan; 101 National Centre for Nuclear Research, Swierk, Poland; 102 Institute of Experimental Physics, Faculty of Physics, University of Warsaw, Warsaw, Poland; 103 Laboratório de Instrumentação e Física Experimental de Partículas, Lisboa, Portugal; 104 Joint Institute for Nuclear Research, Dubna, Russia; 105 Petersburg Nuclear Physics Institute, Gatchina (St. Petersburg), Russia; 106 Institute for Nuclear Research, Moscow, Russia; 107 Institute for Theoretical and Experimental Physics, Moscow, Russia; 108 Moscow State University, Moscow, Russia; 109 P.N. Lebedev Physical Institute, Moscow, Russia; 110 State Research Center of Russian Federation, Institute for High Energy Physics, Protvino, Russia; 111 Faculty of Physics and Vinca Institute of Nuclear Sciences, University of Belgrade, Belgrade, Serbia; 112 Centro de Investigaciones Energéticas Medioambientales y Tecnológicas (CIEMAT), Madrid, Spain; 113 Universidad Autónoma de Madrid, Madrid, Spain; 114 Universidad de Oviedo, Oviedo, Spain; 115 Instituto de Física de Cantabria (IFCA), CSIC-Universidad de Cantabria, Santander, Spain; 116 CERN, European Organization for Nuclear Research, Geneva, Switzerland; 117 Paul Scherrer Institut, Villigen, Switzerland; 118 Institute for Particle Physics, ETH Zurich, Zurich, Switzerland; 119 Universität Zürich, Zurich, Switzerland; 120 National Central University, Chung-Li, Taiwan; 121 National Taiwan University (NTU), Taipei, Taiwan; 122 Chulalongkorn University, Bangkok, Thailand; 123 Cukurova University, Adana, Turkey; 124 Physics Department, Middle East Technical University, Ankara, Turkey; 125 Bogazici University, Istanbul, Turkey; 126 Istanbul Technical University, Istanbul, Turkey; 127 National Scientific Center, Kharkov Institute of Physics and Technology, Kharkov, Ukraine; 128 University of Bristol, Bristol, United Kingdom; 129 Rutherford Appleton Laboratory, Didcot, United Kingdom; 130 Imperial College, London, United Kingdom; 131 Brunel University, Uxbridge, United Kingdom; 132 Baylor University, Waco, USA; 133 The University of Alabama, Tuscaloosa, USA; 134 Boston University, Boston, USA; 135 Brown University, Providence, USA; 136 University of California, Davis, Davis, USA; 137 University of California, Los Angeles, Los Angeles, USA; 138 University of California, Riverside, Riverside, USA; 139 University of California, San Diego, La Jolla, USA; 140 University of California, Santa Barbara, Santa Barbara, USA; 141 California Institute of Technology, Pasadena, USA; 142 Carnegie Mellon University, Pittsburgh, USA; 143 University of Colorado at Boulder, Boulder, USA; 144 Cornell University, Ithaca, USA; 145 Fairfield University, Fairfield, USA; 146 Fermi National Accelerator Laboratory, Batavia, USA; 147 University of Florida, Gainesville, USA; 148 Florida International University, Miami, USA; 149 Florida State University, Tallahassee, USA; 150 Florida Institute of Technology, Melbourne, USA; 151 University of Illinois at Chicago (UIC), Chicago, USA; 152 The University of Iowa, Iowa City, USA; 153 Johns Hopkins University, Baltimore, USA; 154 The University of Kansas, Lawrence, USA; 155 Kansas State University, Manhattan, USA; 156 Lawrence Livermore National Laboratory, Livermore, USA; 157 University of Maryland, College Park, USA; 158 Massachusetts Institute of Technology, Cambridge, USA; 159 University of Minnesota, Minneapolis, USA; 160 University of Mississippi, Oxford, USA; 161 University of Nebraska-Lincoln, Lincoln, USA; 162 State University of New York at Buffalo, Buffalo, USA; 163 Northeastern University, Boston, USA; 164 Northwestern University, Evanston, USA; 165 University of Notre Dame, Notre Dame, USA; 166 The Ohio State University, Columbus, USA; 167 Princeton University, Princeton, USA; 168 University of Puerto Rico, Mayaguez, USA; 169 Purdue University, West Lafayette, USA; 170 Purdue University Calumet, Hammond, USA; 171 Rice University, Houston, USA; 172 University of Rochester, Rochester, USA; 173 The Rockefeller University, New York, USA; 174 Rutgers, the State University of New Jersey, Piscataway, USA; 175 University of Tennessee, Knoxville, USA; 176 Texas A&M University, College Station, USA; 177 Texas Tech University, Lubbock, USA; 178 Vanderbilt University, Nashville, USA; 179 University of Virginia, Charlottesville, USA; 180 Wayne State University, Detroit, USA; 181 University of Wisconsin, Madison, USA

## Abstract

A measurement of the inclusive WW+WZ diboson production cross section in proton–proton collisions is reported, based on events containing a leptonically decaying W boson and exactly two jets. The data sample, collected at $\sqrt{s} = 7~\mbox{TeV}$ with the CMS detector at the LHC, corresponds to an integrated luminosity of 5.0 fb^−1^. The measured value of the sum of the inclusive WW and WZ cross sections is *σ*(pp→WW+WZ)=68.9±8.7 (stat.)±9.7 (syst.)±1.5 (lum.) pb, consistent with the standard model prediction of 65.6±2.2 pb. This is the first measurement of WW+WZ production in pp collisions using this signature. No evidence for anomalous triple gauge couplings is found and upper limits are set on their magnitudes.

The gauge symmetry of the standard model (SM) fixes the triple gauge boson couplings that determine the self-interactions of W and Z bosons. The pair production of vector gauge bosons allows a direct test of the electroweak sector of the SM [1]. Observation of anomalous triple gauge boson couplings would be an indication of physics beyond the SM.

We report the first measurement of WW+WZ diboson production in pp collisions in the semileptonic final state at the Large Hadron Collider (LHC), where one W boson decays leptonically (*ℓν*, with *ℓ*=e,*μ*) while the other boson (W or Z) decays hadronically (*jj*), giving rise to two energetic jets in the final state. Previous measurements in this channel at the Tevatron $\mathrm{p}\bar{\rm p}$ collider include the recent CDF [2] and D0 [3, 4] results. The advantage of reconstructing WW+WZ in the *ℓνjj* decay mode over the purely leptonic final states [5–8] is the larger branching fraction of W and Z bosons to quarks. This advantage is partially offset by the larger backgrounds in the *ℓνjj* channel, coming mainly from W+jets production. In contrast to the fully leptonic decay of WW pairs, the semileptonic process permits a direct measurement of the boson transverse momentum (*p*_T_). The sensitivity of WW production to the WW*γ* coupling and of WW and WZ production at high boson transverse momentum to the WWZ coupling makes these processes particularly useful as a probe of anomalous triple gauge boson couplings.

The data correspond to an integrated luminosity of 5.0±0.1 fb^−1^ collected in 2010 and 2011 with the Compact Muon Solenoid (CMS) detector in pp collisions at $\sqrt{s} = 7~\mbox{TeV}$ at the CERN LHC. The CMS experiment [9] uses a right-handed coordinate system, with the origin at the nominal interaction point, the *x* axis pointing to the center of the LHC ring, the *y* axis pointing up, perpendicular to the plane of the LHC ring, and the *z* axis along the counterclockwise beam direction. The polar angle *θ* is measured from the positive *z* axis and the azimuthal angle *ϕ* is measured in the *x*–*y* plane. The pseudorapidity is defined as *η*=−ln[tan(*θ*/2)]. The central feature of the CMS apparatus is a superconducting solenoid of 6 m internal diameter, providing a magnetic field of 3.8 T. Within the field volume are silicon pixel and strip trackers and several calorimeters. The tracking system covers the range |*η*|<2.5 and provides a track momentum resolution of 1 % at 100 GeV. The lead tungstate crystal electromagnetic calorimeter (ECAL) covers |*η*|<3 with an energy resolution of about $3~\%/\sqrt{E}$, where *E* is in GeV [10]. The brass/scintillator hadron calorimeter (HCAL) covers |*η*|<3.0 with an energy resolution of $100~\%/\sqrt{E}$. The muon system consists of gas-ionization detectors inside and around the steel return yoke, and is capable of reconstructing and identifying muons within |*η*|<2.4. Extensive forward calorimetry complements the coverage provided by the barrel and endcap detectors. The CMS detector is nearly hermetic, allowing for measurements of the missing transverse energy (${E_{\mathrm{T}}^{\text{miss}}}$) in the event. A two-tier trigger system selects the events of interest.

The data were collected with a suite of single-lepton triggers mostly using *p*_T_ thresholds of 24 GeV for muons and 25–32 GeV for electrons. To preferentially select events with on-shell W bosons, the single-electron triggers also require minimum thresholds on ${E_{\mathrm{T}}^{\text{miss}}}$ in the range 0–25 GeV and on the transverse mass *m*_T_ of the electron plus ${E_{\mathrm{T}}^{\text{miss}}}$ system in the range 0–50 GeV. The overall trigger efficiency is about 94 % (90 %) for muon (electron) data, with a small dependence (a few percent) on *p*_T_ and *η*. Simulated events are corrected for the trigger efficiency as a function of lepton *p*_T_ and *η*, and in the case of electrons also as a function of ${E_{\mathrm{T}}^{\text{miss}}}$. Simulated events are used to develop and validate the methods used in the analysis.

The MadGraph5 1.3.30 [11] event generator produces parton-level events with a W boson and up to four partons on the basis of matrix-element (ME) calculations. The ME–parton shower (ME–PS) matching scale *μ* is taken to be 20 GeV [12], and the factorization and renormalization scales are both set to $q^{2} = M_{ \mathrm {W} }^{2} + p_{\mathrm{T}, \mathrm {W} }^{2}$. Samples of $\mathrm {t}\overline {\mathrm {t}}$ and Drell–Yan events are also generated with MadGraph. Single-top production is modeled with powheg 1.0 [13]. Multijet and diboson samples (W W, W Z, Z Z) are generated with pythia 6.422 [14]. pythia provides the parton shower simulation in all cases, with parameters of the underlying event set to the Z2 tune [15]. The set of parton distribution functions used is cteq6ll [16]. A Geant4-based simulation [17] of the CMS detector is used in the production of all Monte Carlo (MC) samples. Multiple proton–proton interactions within a bunch crossing (pileup) are simulated, and the triggers are emulated. All simulated events are reconstructed and analyzed as measured collision events.

Events are selected with one well-identified and isolated lepton (muon or electron), large missing transverse energy, and exactly two high-*p*_T_ jets. Muons are reconstructed within |*η*|<2.1 with the inner tracker and the muon system [18]. Electrons are reconstructed from tracks in the tracker pointing to energy clusters in the ECAL, within |*η*|<2.5, excluding the transition region between the barrel and endcap, 1.44<|*η*|<1.57 [19]. Muons (electrons) are required to have *p*_T_ greater than 25 GeV (35 GeV). The lepton candidates are required to be consistent with having originated from the primary vertex of the event, which is chosen to be the vertex with the highest ∑*p*_T_^2^ of its associated tracks. According to the simulation, this requirement provides the correct assignment for the primary vertex in more than 99 % of the cases in both signal and background events. Charged leptons from W boson decays are expected to be isolated from other activity in the event. The sum of transverse momentum or energy in the tracker, ECAL, and HCAL, within a surrounding cone of $\varDelta R \equiv \sqrt{(\varDelta \eta)^{2}+(\varDelta \phi)^{2}} <0.3$, excluding the lepton itself, is required to be less than 10 % of the measured *p*_T_ of the muon, or less than 5 % of the measured *p*_T_ of the electron. Here *Δη* and *Δϕ* are the differences in pseudorapidity and in azimuthal angle, respectively. To reduce the backgrounds from fully leptonic decays, such as Drell–Yan and electroweak diboson processes, we exclude events in which there is any other loosely identified lepton (with *p*_T_>10 GeV for muons and *p*_T_>20 GeV for electrons) in the event.

Jets are reconstructed from calorimeter and tracker information using a particle-flow technique that combines information from several subdetectors [20]. The anti-*k*_T_ clustering algorithm [21, 22] with a distance parameter of 0.5 is used. Jets that overlap with isolated leptons within *ΔR*=0.3 are not considered. Jet-energy corrections are applied to account for the nonlinear energy response of the calorimeters and for other instrumental effects [23]. These corrections are based on in situ measurements using dijet, *γ*+jet, and Z+jet data samples [24]. Pileup collisions and the underlying event add to the energy of the reconstructed jets. The median energy density from pileup is evaluated in each event and the corresponding energy is subtracted from each jet [25]. In addition, charged tracks that do not originate from the primary vertex are not considered for jet clustering [26]. We verified that these procedures successfully remove the dependence of jet response on the number of interactions in a single event. A jet-quality requirement, primarily based on the energy balance between charged and neutral hadrons in a jet, is applied to remove poorly reconstructed jets. Only events containing exactly two jets with *p*_T_>35 GeV and within |*η*|<2.4 are selected for the analysis. To reduce contamination from $\mathrm {t}\overline {\mathrm {t}}$ background, events are discarded if one or more jets pass high-efficiency b-quark jet identification criteria based on the presence of a secondary vertex within the jet [27]. An accurate ${E_{\mathrm{T}}^{\text{miss}}}$ measurement is essential to distinguish the W signal from multijet backgrounds and to reconstruct the full event kinematics of the WW system. We use ${E_{\mathrm{T}}^{\text{miss}}}$ measured in the event using the full particle-flow reconstruction [28] and require ${E_{\mathrm{T}}^{\text{miss}}}>25$ (30) GeV for the muon (electron) channel. To reduce the background from processes that do not contain W→*ℓν* decays, we require that the transverse mass of the W candidate exceed 30 GeV (50 GeV) in muon (electron) data [29].

We measure the dijet mass (*m*_*jj*_) distribution, as shown in Fig. 1(a). The relative contributions of the known SM processes are determined using an unbinned maximum-likelihood fit over the mass range 40–150 GeV. The fit is performed separately for the muon and electron channels since their background compositions differ. Table 1 lists the SM processes included in the fit. The normalization of the diboson WW+WZ contribution is a free parameter. The normalizations of the background components are allowed to vary within Gaussian constraints around their central values. For multijet events, this central value is obtained from an independent two-component fit to the ${E_{\mathrm{T}}^{\text{miss}}}$ distribution which determines the corresponding fraction in the data [29]. The fit uncertainty is used as a constraint on the multijet contribution. The central values for all other processes are obtained from next-to-leading-order (NLO) or higher-order calculations, and the constraints are taken from the theoretical uncertainties listed in Table 1. With the exception of multijet production, the shape of the *m*_*jj*_ distribution for all processes is obtained from simulation. Multijet events contribute to the total background when jets are misidentified as isolated leptons. Their *m*_*jj*_ shape can be derived from data events with lepton candidates that fail the isolation requirements. The fluctuations in the shapes and yields of subleading backgrounds have a minor impact on the overall fit.

**Fig. 1:**
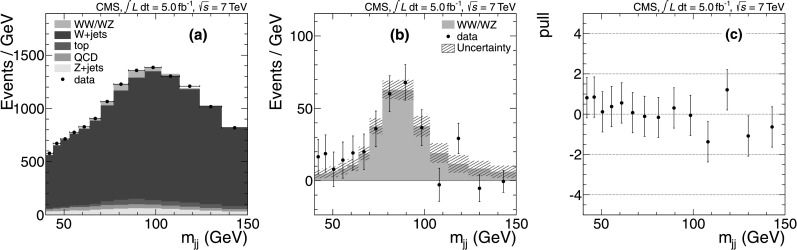
(**a**) Distribution of the dijet invariant mass in data, with the binning chosen based on the resolution and fit projections of the relevant components overlaid. (**b**) The dijet invariant mass after subtraction of all components except the electroweak WW+WZ processes. The *error bars* represent the statistical uncertainties and the *hatched bands* represent the systematic uncertainties. (**c**) The normalized residual or pull: (data−fit)/(fit uncertainty) (Color figure online)

**Table 1:** Treatment of background *m*_*jj*_ shapes and normalizations in a fit to the data. The cross section values are calculated with the programs cited on the corresponding rows. The background normalizations are constrained to Gaussian distributions with the listed central values and widths. The treatment of multijet events is described in the text

Process	Shape	Constraint on normalization
Diboson (WW+WZ)	MC	Unconstrained
W+jets	MC	31.3 pb±5 % (NLO) [30]
$\mathrm {t}\overline {\mathrm {t}}$	MC	163 pb±7 % (NLO) [31]
Single top	MC	85 pb±5 % (NNLL) [32–34]
Drell–Yan+jets	MC	3.05 pb±4.3 % (NNLO) [35]
Multijet (QCD)	data	${E_{\mathrm{T}}^{\text{miss}}}$ fit in data

The *m*_*jj*_ spectrum of the dominant W+jets component is described using the shape from MadGraph simulation after taking into account the uncertainties due to the factorization and renormalization scale (both equal to *q*) and ME–PS matching scale *μ* [36]: 
1 where $\mathcal{F}_{\text{W+jets}}$ denotes the *m*_*jj*_ shape from simulation. The parameters *μ*_0_ (*μ*′) and *q*_0_ (*q*′) correspond to the default (alternative) values of *μ* and *q*, respectively. The parameters *α* and *β* are free to vary during the fit and remain within the physical ranges (0≤*α*,*β*≤1 and 1−*α*−*β*≥0). We take *μ*′=2*μ*_0_ or 0.5*μ*_0_ (*q*′=2*q*_0_ or 0.5*q*_0_), depending on which alternative sample provides a better fit to the data. Thus, the fit probes variations of a factor of two in both *μ* and *q* (with the corresponding shape fluctuations accounted for when setting exclusion limits).

Figure 1(a) shows the observed *m*_*jj*_ distribution for both channels combined, together with the fitted projections of the contribution of various SM processes. Figure 1(b) shows the same distribution after subtracting all SM contributions from data except for WW+WZ events. Figure 1(c) shows the pull distribution, i.e. the normalized residual defined as (data−fit)/(fit uncertainty), where the fit uncertainty is computed at each data point by propagating the uncertainty in the normalization coefficients. The yields of various SM components, as determined by the fit, are reported in Table 2.

**Table 2:** Event yields determined from a maximum-likelihood fit to the data. The total uncertainty is computed using the full covariance matrix. Owing to a higher kinematic threshold the product of acceptance × efficiency is smaller for the electron channel. The term $\mathcal{A}\varepsilon$ includes W and Z branching fractions [37]

Process	Muon channel	Electron channel
Diboson (WW+WZ)	1900±370	800±310
W plus jets	67380±590	31640±850
$\mathrm {t}\overline {\mathrm {t}} $	1660±120	950±70
Single top	650±30	310±20
Drell–Yan+jets	3610±160	1410±60
Multijet (QCD)	300±320	4190±870
Data	75419	39365
Fit *χ* ^2^/*N*_dof_ (probability)	9.73/12 (0.64)	5.30/12 (0.95)
Acceptance × efficiency ($\mathcal{A}\varepsilon$)	(5.15±0.24)×10^−3^	(2.63±0.12)×10^−3^
Expected WW+WZ yield from simulation	1700±60	870±30

In order to ensure robustness against fit parameters and to account for corresponding biases we validate the fit procedure by performing pseudo-experiments. In each experiment, we generate the *m*_*jj*_ pseudo-data for the SM processes, taking into account the correlations between the yields, and then perform a fit to each pseudo-data *m*_*jj*_ distribution. The results for both the muon and electron channels indicate that there is a small bias (−8.6 % and −6.6 %) in the WW+WZ yield, corresponding to less than 0.4 standard deviations, and that the fit slightly overestimates the uncertainty on the yield. These effects are corrected for in the final result. The validation procedure shows that biases in all background yields and errors are small. The fit results for the background components are statistically consistent with the expectations, with the exception of W+jets, where 11 % fewer events for muons and 15 % fewer events for electrons, compared to the expectation, are observed. Overall, the approach produces a high quality model of the data (Fig. 1(a)), where the pull distribution is consistent with 0 (Fig. 1(c)), and allows us to extract the diboson peak (Fig. 1(b)).

Systematic uncertainties arising from the jet energy are estimated from W bosons decaying hadronically in a sample of semileptonic $\mathrm {t}\overline {\mathrm {t}}$ events. The mean and resolution of the reconstructed dijet mass distribution in data agree to within 0.6 % of the expectations from simulation (this discrepancy is accounted for as an explicit systematic uncertainty), with negligible effect on acceptance. A small difference in ${E_{\mathrm{T}}^{\text{miss}}}$ resolution [28] between data and simulation affects the signal acceptance at the 0.5 % level. Further systematic uncertainties on the signal yield are due to the uncertainty on the trigger efficiency in data (1 %), and on the lepton reconstruction and selection efficiencies (2 %) [29]. The uncertainty due to the b-jet veto is negligible. The uncertainty in the luminosity measurement is 2.2 % [38]. The uncertainty in acceptance arising from theoretical uncertainties (evaluated using MadGraph and mcfm samples), including parton distribution functions and additional jet rejection, is 4 %.

As listed in Table 2, we observe 2700±340 (stat.)±360 (syst.) WW+WZ events, in agreement with the SM expectation. This result corresponds to a significance of 8.8 standard deviations when computed using a simple likelihood ratio [39], where the background yield uncertainties (Table 2) and errors on *α*, *β* (Eq. (1)) are fixed to their fitted values. Using the profile likelihood ratio [39], where these parameters are allowed to vary, the significance becomes 4.3 standard deviations. We compute the WW+WZ total cross section as $\sigma = N_{\text{Sig}}/ (\mathcal{A} \, \varepsilon \, {\mathcal{L}})$, where *N*_Sig_ is the number of extracted signal events, $\mathcal{A}$ is the signal acceptance corrected for the branching fractions, *ε* is the overall efficiency for event selection, and ${\mathcal{L}}$ is the integrated luminosity. In the acceptance calculation we assume the SM value for the WW to WZ production ratio. The values of *N*_Sig_ and $\mathcal{A}\varepsilon$ are given in Table 2 separately for the muon and electron channels. Combining the results from the muon and electron channels, we obtain *σ*(pp→WW+WZ)=68.9±8.7 (stat.)±9.7 (syst.)±1.5 (lum.) pb, which is in agreement with the NLO prediction [40], 65.6±2.2 pb, that includes the contribution from gg→WW. The total cross section computed in the muon channel, 73.8±15.1 pb, is consistent with that obtained in the electron channel, 60.8±21.5 pb, where the statistical and systematic uncertainties have been combined in quadrature.

Measurements of electroweak diboson production can be translated into measurements of gauge boson self-couplings, which are among the most fundamental aspects of the SM. At the leading order, only *s*-channel $\mathrm{q}\overline{\mathrm{q}}$ annihilation diagrams have a three-boson vertex involving WW*γ* and WWZ couplings in WW production, and WWZ coupling in WZ production. Physics beyond the SM can modify these couplings, leading to observable differences in the cross section and the kinematic distributions [41]. We search for anomalous triple gauge couplings (aTGCs) using an effective Lagrangian described by the following HISZ (Hagiwara, Ishihara, Szalapski, Zeppenfeld) parametrization without form factors [1]: *λ*_*γ*_=*λ*_*Z*_=*λ*, $\varDelta {\kappa_{Z}} = \varDelta {g_{1}^{Z}}-\varDelta {\kappa_{\gamma}} \cdot \tan^{2}\theta_{\mathrm{W}}$. We use the dijet *p*_T_ distribution (with most of the discriminating power coming from the last bin), shown in Fig. 2, as the observable after requiring 75 GeV<*m*_*jj*_<95 GeV to enhance signal purity. The dependence of the distribution on specific aTGCs is modeled by reweighting the pythia simulation of WW+WZ to the mcfm [30] NLO predictions. We account for systematic uncertainties arising from luminosity, signal selection efficiency (via comparisons to mcfm samples employing alternate choices of PDFs as well as factorization and renormalization scales), signal shape, and from the normalization and shape of the SM processes. We find no evidence for aTGCs. Given the tight bound on the parameter $\varDelta {g_{1}^{Z}}$ [37], we assume the SM value ($\varDelta {g_{1}^{Z}}=0$) and set limits on the two parameters *λ* and *Δκ*_*γ*_. Exclusion limits at 95 % confidence level (CL) in the two-dimensional *λ*-*Δκ*_*γ*_ plane, computed using the modified frequentist CL_*S*_ [39, 42] technique with profile likelihood as the test statistic, are shown in Fig. 3. The limit setting procedure combines fit results from muon and electron channels We obtain the following one-dimensional observed 95 % CL limits assuming the SM value for the other parameter: −0.038<*λ*<0.030, −0.11<*Δκ*_*γ*_<0.14. These limits are competitive with, and in some cases improve upon, the sensitivity of the combined LEP experiments listed in Refs. [37, 43–46]. The ATLAS Collaboration recently reported limits in the fully leptonic channel for WZ [7] and WW [8] production. Limits obtained from fully leptonic channels are weaker due to the smaller branching fractions.

**Fig. 2:**
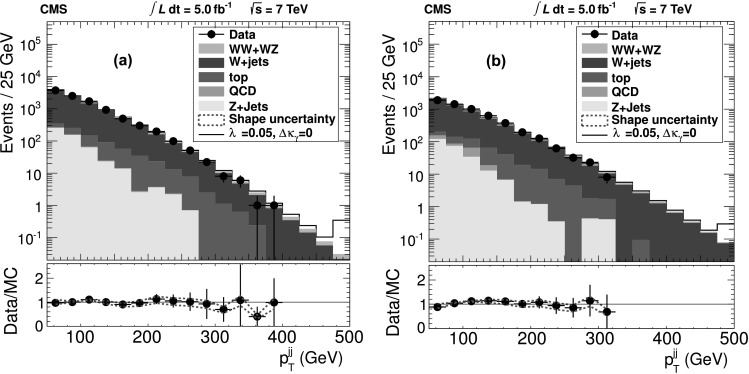
Dijet *p*_T_ distributions for (**a**) the muon and (**b**) the electron channels after full selection and with the requirement 75 GeV<*m*_*jj*_<95 GeV. The stacked histogram shapes are taken from simulation or, where applicable, from data-driven estimates. They are normalized according to the fit to the observed *m*_*jj*_ spectrum in data. Below we show the Data/MC ratio with the (*dashed*) *red lines* corresponding to the shape uncertainty. The last bin includes the overflow (Color figure online)

**Fig. 3:**
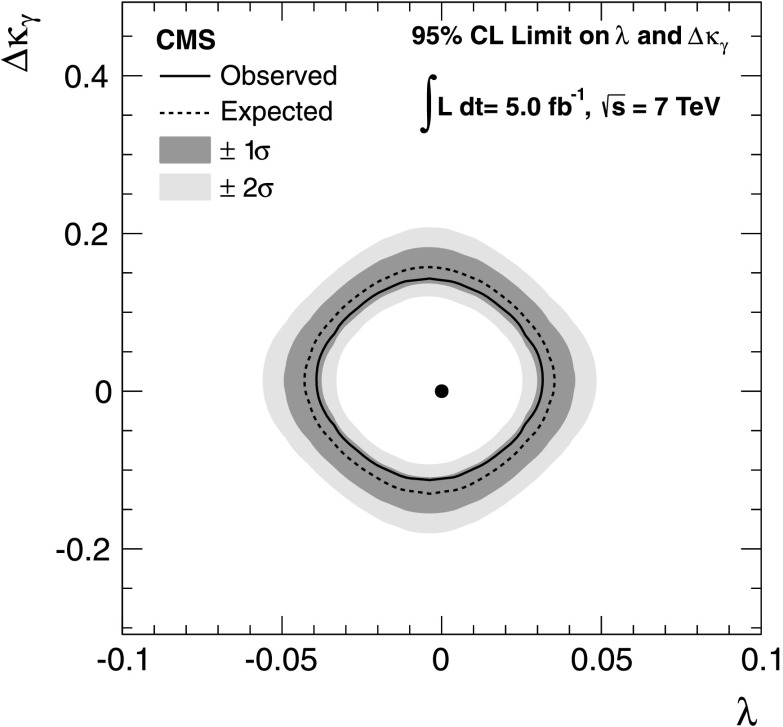
Observed (*solid*) and expected (*dashed*) exclusion limits at 95 % CL for anomalous triple gauge couplings. The *dark green* and *light yellow* bands correspond to the one and two sigma intervals, respectively, in the expected limit distribution. The SM expectation is shown by the *solid dot* (Color figure online)

In summary, a measurement of the sum of the inclusive WW and WZ production cross sections has been performed using events containing a leptonically decaying W and two jets. The measured value for the cross section is *σ*(pp→WW+WZ)=68.9±8.7 (stat.)±9.7 (syst.)±1.5 (lum.) pb, which is consistent with the SM prediction. This is the first measurement of WW+WZ production in pp collisions using this signature. No evidence for anomalous triple gauge couplings is found, and limits are set on their magnitudes.
